# Increased light penetration due to ultrasound-induced air bubbles in optical scattering media

**DOI:** 10.1038/s41598-017-16444-9

**Published:** 2017-11-23

**Authors:** Haemin Kim, Jin Ho Chang

**Affiliations:** 1 Department of Biomedical Engineering, Sogang University, 35 Baekbeom-ro, Mapo-gu, Seoul, 04107 South Korea; 2 Department of Electronic Engineering, Sogang University, 35 Baekbeom-ro, Mapo-gu, Seoul, 04107 South Korea

## Abstract

Light is an attractive tool for high spatial- and contrast-resolution imaging, highly sensitive molecular imaging, and target-selective therapy, and it does not exhibit the risks associated with ionizing radiation. The main limitation of using light in clinical applications is its superficial imaging and therapeutic depth caused by high optical scattering in biological media. Here, we demonstrate that the scattering and thus defocusing of the incident light can be alleviated when simultaneously delivered ultrasound generates air bubbles in the pathway of the incident light, thus increasing the light penetration. The bubbles are temporally induced by ultrasound with an intensity that is sufficiently low to avoid tissue damage and act as a Mie scattering medium in which light is scattered predominantly in the forward direction. The change in the optical scattering property caused by the ultrasound is undone after cessation of the insonification. From the results, it is expected that this proposed method will open a new route for overcoming the limitations of current optical imaging and therapeutic techniques.

## Introduction

Light delivery into a deep-lying target region is a major challenge in optical imaging and therapy. This challenge is primarily due to optical scattering in biological media because the scattering is dominant over the absorption in biological media and thus determines the ballistic regime and the transport mean free path length^1^. To overcome this hurdle, several optical focusing methods based on a guide star have been proposed^2^; ultrasound^3–5^ and photoacoustic^6–8^ signals have been used to generate a guide star non-invasively in non-optical approaches. In addition, it was demonstrated that microbubbles administered to a blood vessel can be used to form a guide star^9^. These methods involve the acquisition of information about a guide star and/or iterative wavefront shaping for light focusing with the help of the guide star, which is burdensome for real-time operation *in vivo* ^2^. Additionally, to achieve optimal performance, it is desirable that an ultrasound transducer is placed perpendicular to the light propagation direction. However, this configuration may be an obstacle to the implementation of these methods for clinical applications, especially for endoscopic optical imaging and therapy, in which parallel alignment between the propagation directions of light and the interrogated direction of an ultrasound transducer is preferable for the realization of a compact probe.

Optical scattering is generally categorized as either Rayleigh scattering or Mie scattering, depending on the ratio of the radius of a spherical particle to light wavelength. Rayleigh scattering occurs in all directions when the particle size is much smaller than the light wavelength. In contrast, in the Mie scattering regime, which corresponds to when the particle size is comparable to the light wavelength, incident light is predominantly scattered in the forward direction^10^. Therefore, all other conditions being equal, the light penetration depth is longer in Mie scattering media than in Rayleigh scattering media. Biological tissues feature both scattering regimes, with each case exhibiting different contributions of Rayleigh and Mie scattering; Rayleigh scattering significantly increases the reduced scattering coefficients of biological tissues when the light wavelength is less than 800 nm^11^. This fact implies that optical scattering can be significantly reduced if biological tissue can be changed to a Mie scattering dominant medium.

Ultrasound can induce an increase in the local temperature around its focal area in biological tissues. When the local temperature reaches a certain level (i.e., above 60 °C), thermal damage to the tissue occurs in the focal area^12^; this has been used for noninvasive cancer treatment called high-intensity focused ultrasound (HIFU) surgery. Interestingly, it was observed that air bubbles are generated within the focal area due to the rapid change in the acoustic pressure, even when the ultrasound increases the local temperature to a value that does not damage the tissue at all^13–15^. In this paper, we demonstrate that air bubbles temporally induced by insonification parallel to the light incident direction can be used to increase light penetration in biological tissues. The rationale behind this phenomenon is that the air bubbles act as a Mie scattering medium. Because light is scattered predominantly in the forward direction in the Mie scattering regime, the light propagating through the bubble cloud is less spread and thus is less defocused, compared with the delivery of light only. This concept was verified by tissue-mimicking phantom and *ex vivo* chicken breast tissue experiments. Additionally, it was ascertained from fluorescence imaging experiment that the simultaneously transmitted ultrasound is sufficiently high to form air bubbles but low enough to avoid tissue damage.

## Results

**Figure 1:**
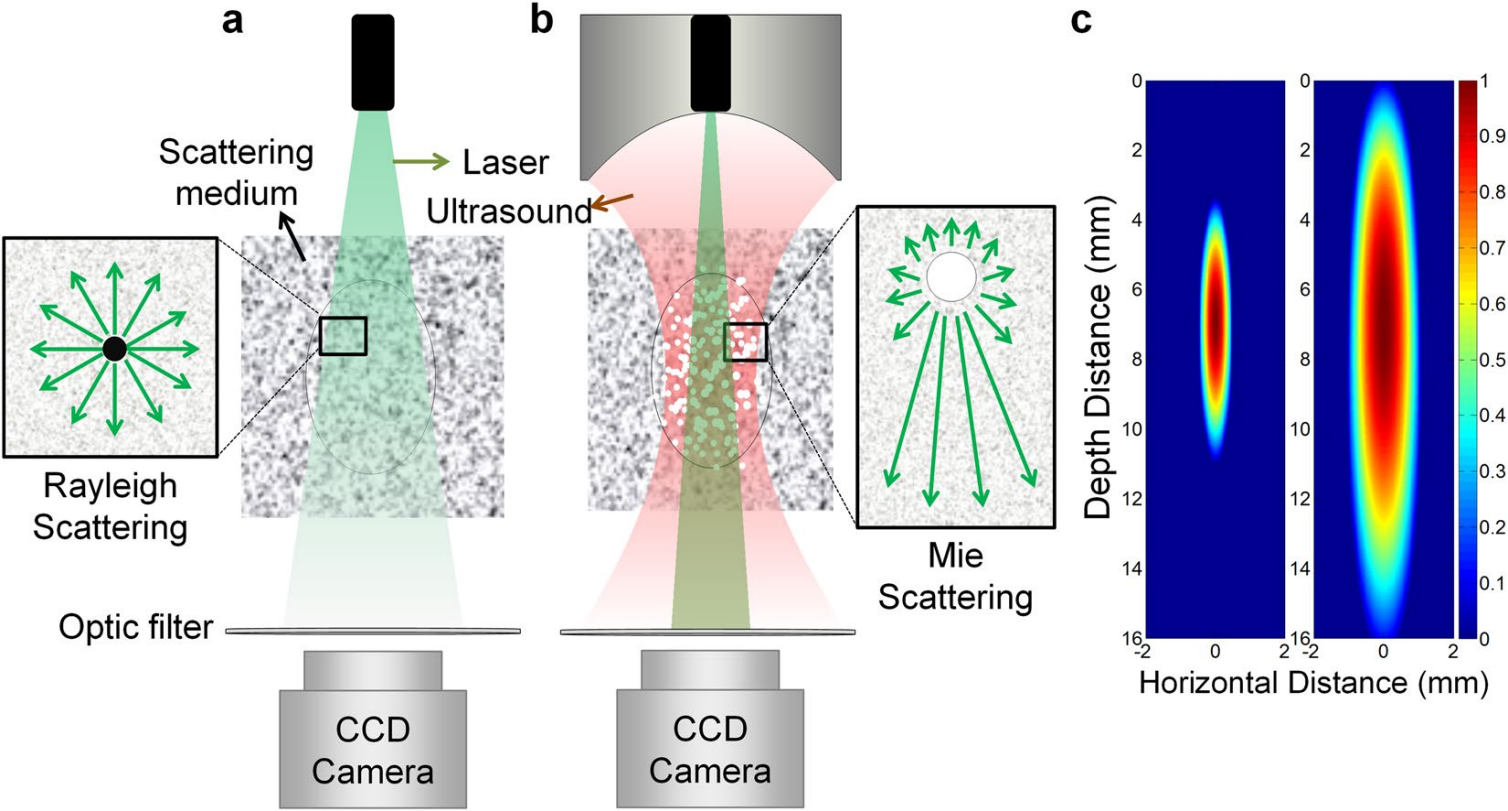
Conceptual illustration of increased light penetration due to ultrasound-induced air bubbles in scattering media. (**a**) Light is spread, and its intensity is decreased as it travels through a Rayleigh scattering medium in which light scattering occurs in all directions. (**b**) Ultrasound generates air bubbles within its focal area, and the incident light experiences Mie scattering in the bubble cloud; thus, its scattering occurs predominantly in the forward direction. As a result of this phenomenon, the light spread is decreased, resulting in increased light penetration depth. (**c**) Simulated ultrasound beam profile contributing to bubble generation. The area covered by the beam profile becomes much larger along the depth direction than along the horizontal direction as the ultrasound intensity increases, i.e., the left panel to the right, which means that the size and population of the bubble cloud in light pathway increase with the ultrasound intensity.

Figure 1a shows that light traveling through a Rayleigh scattering medium is scattered in all the directions, thus causing the light to be spread over the medium and its intensity to be significantly decreased. When ultrasound with a certain level of intensity is transmitted into the medium, air bubbles are generated within its focal area (Fig. 1b). If light propagates through the air bubbles, the light experiences Mie scattering rather than Rayleigh scattering, thus being scattered predominantly in the forward direction. This implies that the scattering and thus defocusing of the incident light can be alleviated when a simultaneously delivered ultrasound generates air bubbles in the pathway of the incident light, thus increasing the light penetration. Although ultrasound energy distribution is determined by the configuration and operating frequency of an ultrasound transducer^16^, an effective area in which air bubbles are induced increases as ultrasound intensity increases; the increase in area is much larger in the depth direction than in the horizontal direction (Fig. 1c). This means that it is possible to increase the size and population of the bubble cloud in light pathway by adjusting ultrasound intensity, which results in minimizing the scattering and defocusing of incident light.

**Figure 2:**
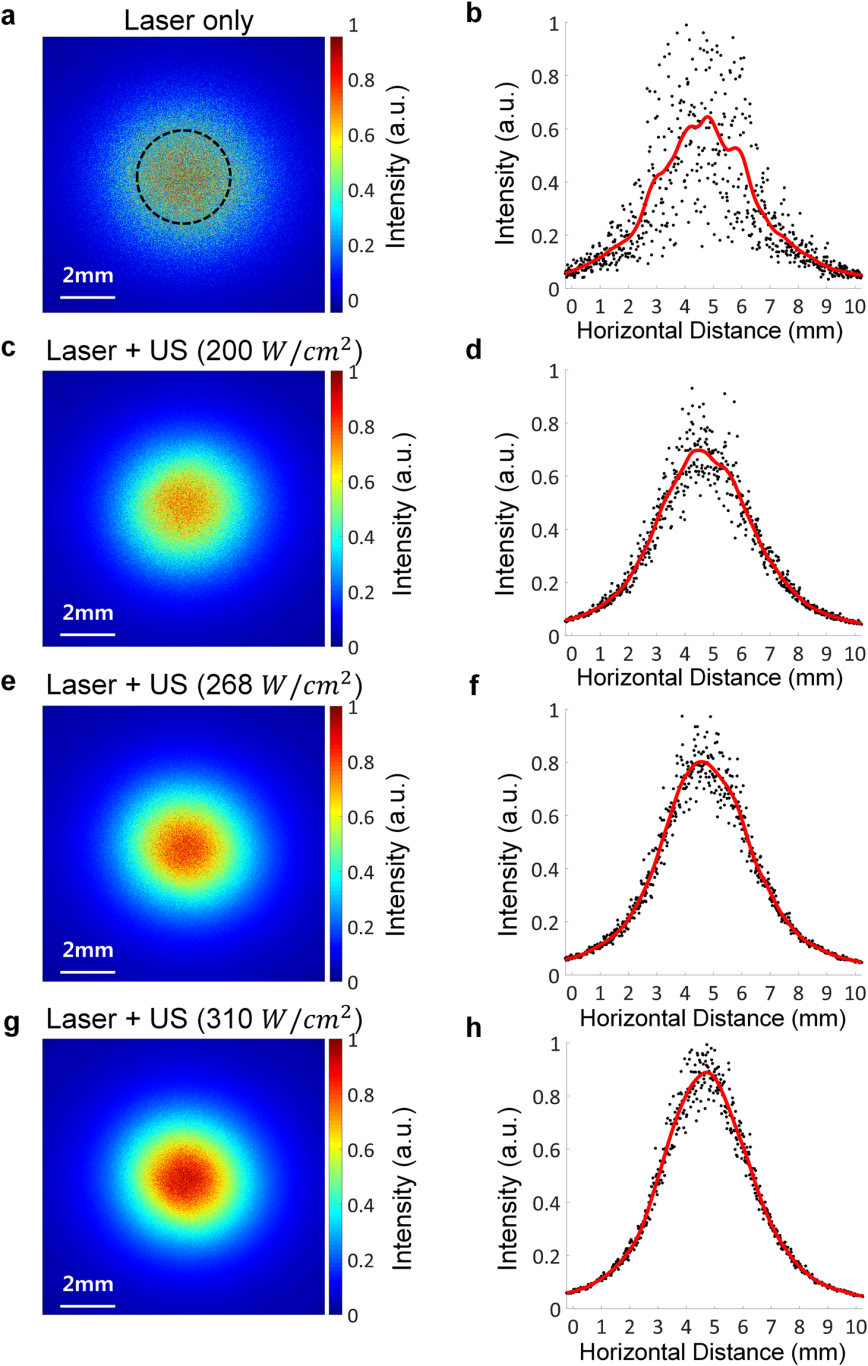
Measurement of light intensity distribution on the tissue-mimicking phantom with a thickness of 8 mm. (**a**) Light intensity distribution measured at the end of the phantom by a CCD camera when laser irradiation only was delivered. (**b**) The distribution along the horizontal axis (dots) and the corresponding smoothing spline fitting curve (red line) are shown. (**c**) to (**h**) are light intensity distributions when both laser and ultrasound (US) irradiation with the intensity of 200 W/cm^2^ in (**c**) and (**d**), 268 W/cm^2^ in (**e**) and (**f**), and 310 W/cm^2^ in (**g**) and (**h**) were delivered into the medium. The ROI within which the average normalized light intensity was measured is indicated by a black dashed circle.

The performance of the proposed method was verified by phantom test. For this, either laser only or both laser and ultrasound were delivered into a tissue-mimicking phantom with a thickness of 8 mm, and the light intensity was measured at the end of the phantom using a charge-coupled device (CCD) camera. Three different ultrasound intensities of 200, 268, and 310 W/cm^2^ were used; these led to the increases in the local temperature of the ultrasound focal area: 1, 3, and 5 °C, respectively. These small temperature increases did not generate coagulation in the focal area^17^. Additionally, the light penetration depth would be significantly decreased if tissue coagulation is induced in the traveling path of the light because tissue coagulation leads to an increase in the optical scattering by more than 25 times in the case of chicken breast tissue^18^. The laser with a Gaussian beam profile and a full-width at half-maximum (FWHM) of 3.4 mm was significantly scattered and defocused after traveling into the phantom when the laser was solely delivered into the medium (Fig. 2a); the light intensity fluctuated strongly along the horizontal axis, and its initial beam profile disappeared (Fig. 2b). The average normalized intensity within a FWHM of 3.4 mm was measured to be 0.520 ± 0.026. In contrast, the scattering and defocusing effect was alleviated when ultrasound was transmitted in conjunction with the laser irradiation (Fig. 2c to h). The degree of the improvement depended on the ultrasound intensity. The fluctuations in the light intensity became weaker and the light intensity distribution became close to a Gaussian shape as the ultrasound intensity was increased. As the ultrasound intensity was increased to 200, 268, and 310 W/cm^2^, the average normalized intensity changed to 0.597 ± 0.029, 0.623 ± 0.020, and 0.649 ± 0.037, respectively, corresponding to the mean enhancement ratios of 14.81, 19.81, and 24.81%. Additionally, the light intensity distribution in the case of 310 W/cm^2^ exhibited a Gaussian shape, and its FWHM was measured to be 4.23 mm.

**Figure 3:**
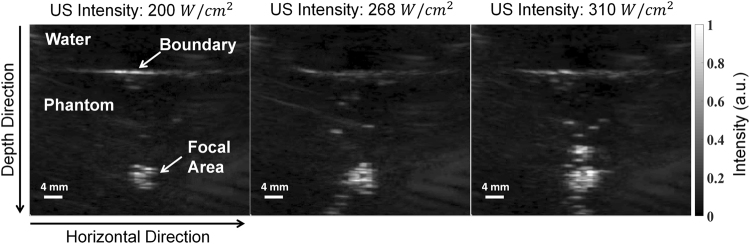
Ultrasound images of the air bubbles induced by the incident ultrasound in the tissue-mimicking phantom.

The reason for the increase in light intensity as ultrasound intensity increases can be elucidated through the examination of the air bubbles induced by the incident ultrasound. Figure 3 demonstrates that the bubble cloud grew with increasing ultrasound intensity. In particular, the growth rate was much larger in the depth direction than in the horizontal direction; the maximum dimensions of the bubble cloud were 5.4 and 5.6 mm in the depth and horizontal directions, respectively, at 200 W/cm^2^, 5.9 and 6.5 mm at 268 W/cm^2^, and 12.5 and 7.3 mm at 310 W/cm^2^. We note that in all the cases, the size of the bubble cloud was larger than the laser beam diameter of 3.4 mm. This result implies that the Mie scattering regime increases along the depth direction with increased ultrasound intensity, thus leading to less defocusing of the incident laser beam.

**Figure 4:**
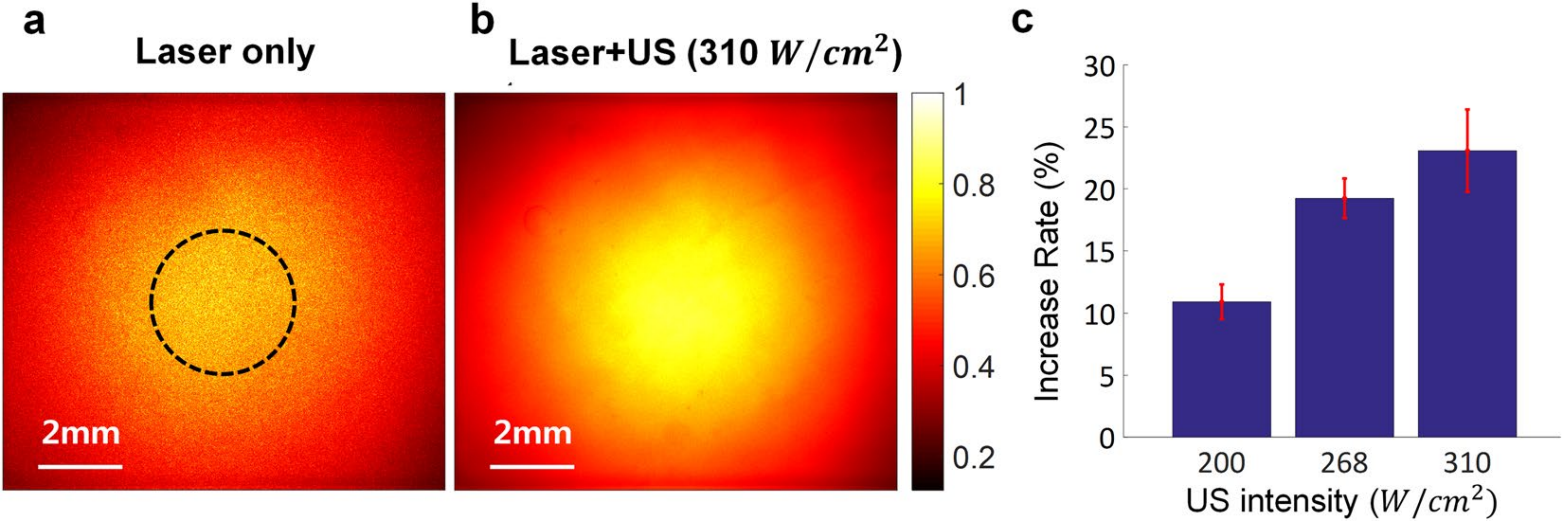
Measurement of light intensity distribution on the *ex vivo* chicken breast with a thickness of 7 mm. (**a**) Light intensity distribution measured at the end of the chicken breast by a CCD camera when laser irradiation only was delivered and (**b**) both laser and ultrasound (US) with an intensity of 310 W/cm^2^ were delivered into the medium. (**c**) Average light intensities at each ultrasound intensity and the rate of intensity increase from the intensity measured when only the laser was transmitted. The ROI within which the average normalized intensity was measured is indicated by a black dashed circle.

**Figure 5:**
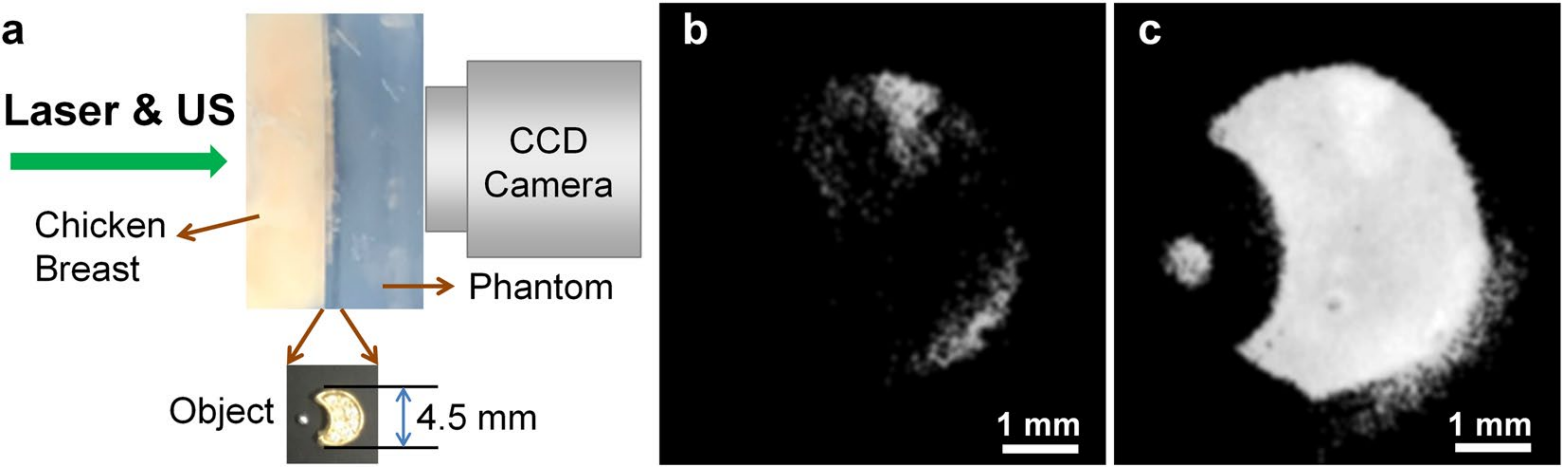
Fluorescence image of an object embedded in between *ex vivo* chicken breast and a tissue-mimicking phantom. (**a**) Illustration of sample arrangement. Fluorescence images of the object acquired after (**b**) laser delivery only and (**c**) simultaneous transmission of laser and ultrasound.

The same phenomena were observed in *ex vivo* chicken breast with a thickness of 7 mm. The average normalized light intensity was 0.68 ± 0.047 in the case of laser delivery only, and this was increased by 10.8, 19.2, and 23.0% when the intensity of the simultaneously transmitted ultrasound was increased to 200, 268, and 310 W/cm^2^, respectively (Fig. 4). Additionally, the examination of the light intensity distribution indicated that the amount of defocusing decreased as the ultrasound intensity increased, consistent with the results of the phantom experiment (refer to Fig. S2). The benefit of the simultaneous transmission of ultrasound and light is more obvious when acquiring the fluorescence image of a complex object embedded in between *ex vivo* chicken breast and a tissue-mimicking phantom (Fig. 5). We patterned the shape of a crescent moon and a circle in the phantom and filled the pattern with fluorescent dyes. *Ex vivo* chicken breast with a thickness of 8 mm was placed in front of the pattern as shown in Fig. 5a. Either laser only or both laser and ultrasound were transmitted to the target through the chicken breast tissue, and fluorescence emitted from the fluorescent dye in the pattern was measured at the end of the phantom. The pattern was clearly visible in the fluorescence image acquired after delivering laser and ultrasound with an intensity of 310 W/cm^2^ at the same time (Fig. 5c), whereas the pattern did not clearly appear in the case of laser delivery only (Fig. 5b).

**Figure 6:**
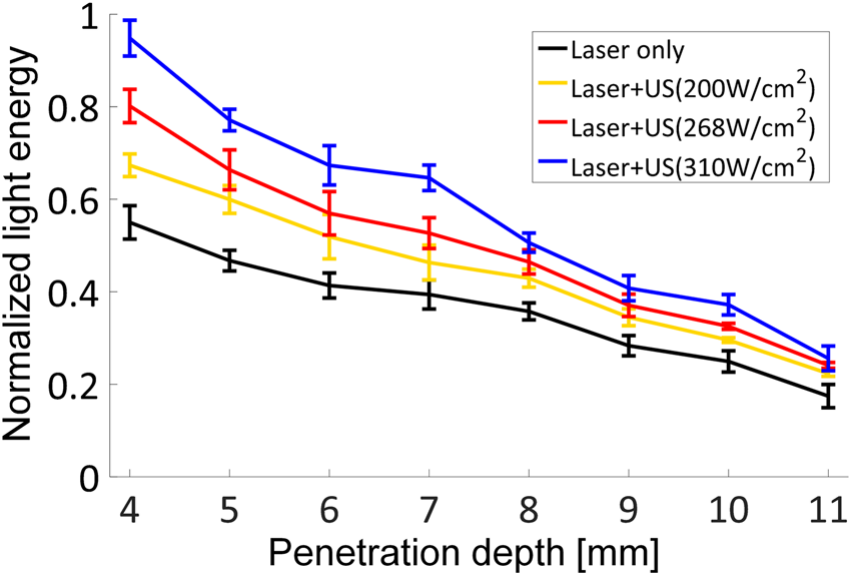
Plot of the normalized light energy measured in the tissue-mimicking phantoms of different thickness.

In addition to the reduction of defocusing, it was ascertained that the proposed method facilitates increasing the depth of light penetration. For this, the tissue-mimicking phantoms with different thicknesses were prepared; the thickness was changed from 4 to 11 mm in 1-mm increments. The light energy was measured at the end of the phantoms using an optical spectrometer (Fig. 6). When only laser was transmitted, the normalized light energy was 0.550 ± 0.036 at a depth of 4 mm and gradually decreased to 0.394 ± 0.031 at 7 mm, 0.358 ± 0.018 at 8 mm, and 0.175 ± 0.025 at 11 mm. After simultaneous delivery of laser and ultrasound with an intensity of 310 W/cm^2^, however, this value increased to 0.948 ± 0.039 (an increase rate of 72.4%), 0.646 ± 0.028 (an increase rate of 64.0%), 0.506 ± 0.021 (an increase rate of 41.5%), and 0.261 ± 0.017 (an increase rate of 49.5%) at the respective depths. Based on the results, it can be concluded that the proposed method is able to increase the penetration depth of light although the rate of increase depends on ultrasound intensity and desired light energy.

## Discussion

It has been shown that ultrasound energy can generate air bubbles within the focal area and thus turn biological tissue into a Mie scattering dominant medium. This ultrasound energy inevitably increases the local temperature of the focal area (i.e., up to 5 °C in this study). In fact, optical scattering is also influenced by the medium temperature. For example, with increasing temperature, the scattering coefficients of epidermis, subcutaneous tissue, and lecithin membrane decrease, in contrast to the increase observed in the case of dermis^19–21^. However, the *ex vivo* experimental results have demonstrated that the local temperature rise due to the incident ultrasound does not (or is less likely to) play a role of reducing the optical scattering and beam defocusing because the optical scattering in a tissue largely composed of protein, such as chicken breast, increases with increased temperature of the medium^20^. Based on the results, therefore, it can be concluded that the ultrasound-assisted light penetration increase is not related to the local temperature increase.

**Figure 7:**
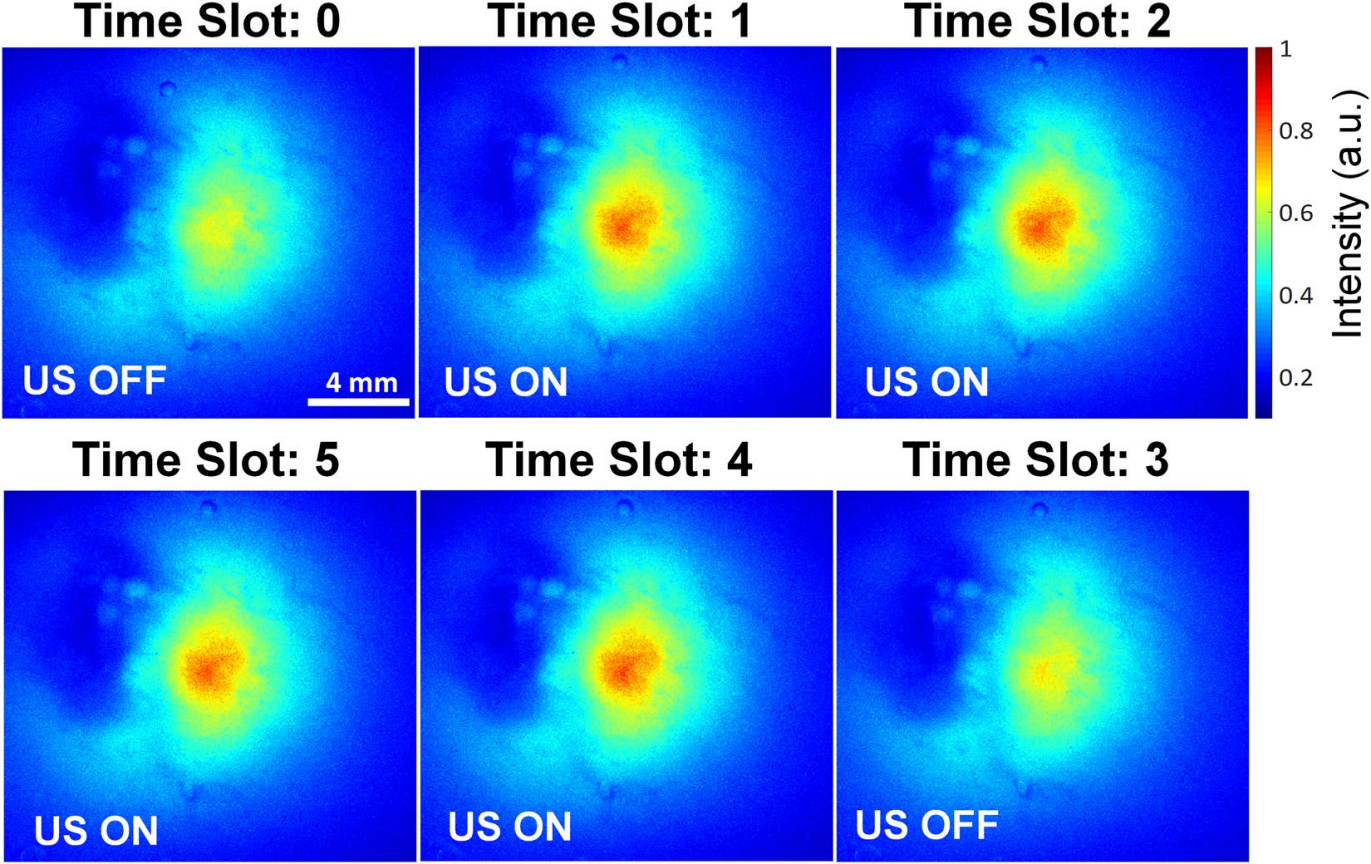
Change in the intensity of fluorescence emitted from the fluorescent dye. The fluorescence emission in response to the interaction between the incident laser and fluorescent dye (Alexa Fluor 660 dye) increased when both the laser and the ultrasound with an intensity of 310 W/cm^2^ were simultaneously delivered into the chicken breast containing the fluorescent dye at a depth of 7 mm.

The amount of ultrasound intensity used in this study did not generate tissue coagulation at the focal area, which was confirmed by detecting the fluorescence emitted from a fluorescent dye injected into an *ex vivo* chicken breast at a depth of 7 mm (Fig. 7). The fluorescence emission induced solely by the incident laser exhibited a peak normalized intensity of 0.71, whereas this value was increased when ultrasound with an intensity of 310 W/cm^2^ was delivered at the same time; the increase rate was 21.1% at Time Slots 1 and 2. Upon the cessation of the ultrasound transmission, this fluorescence intensity was restored to 0.72, which was similar to the initial value (see Time Slot 3 in Fig. 6). When both ultrasound and laser were delivered again, the fluorescence intensity increased from the initial value to 0.88 and 0.85 (corresponding to increases of 24 and 20%) at Time Slots 4 and 5, respectively; these values are similar to the values for Time Slots 1 and 2. This result implies that the transmitted ultrasound temporarily changed the light scattering property of the tissue in the laser pathway to that of a predominantly Mie scattering medium without either tissue damage or permanent alteration of the tissue properties. Otherwise, the regression and re-augmentation of light intensity cannot be controlled by ultrasound.

**Figure 8:**
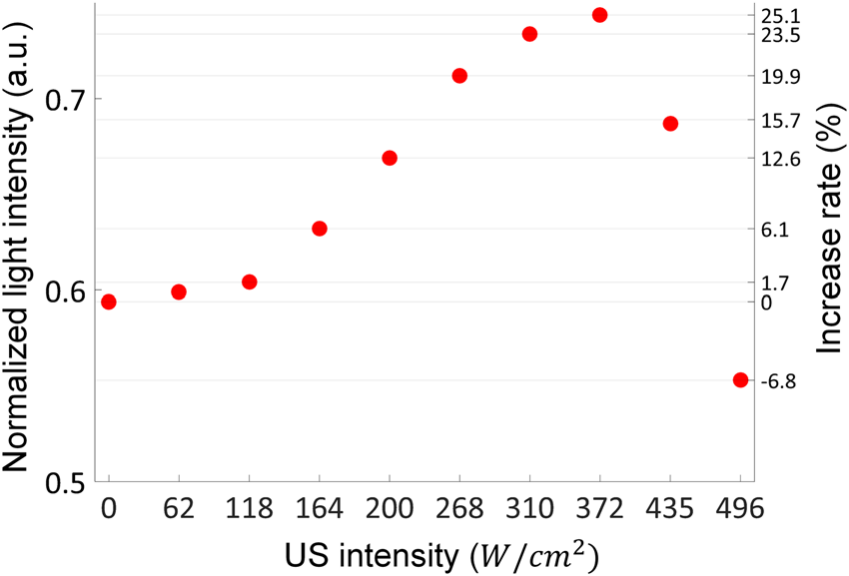
Change in the average normalized light intensity measured in the tissue-mimicking phantom as the ultrasound intensity increases.

Figure 8 shows how the permanent change of a medium (e.g., coagulation) influences the light intensity enhancement by the proposed approach. In the tissue-mimicking phantom, the bubble cloud began to be induced at an ultrasound intensity of 164 W/cm^2^ (Fig. S3), and the maximum rate of increase in light intensity was 25.1% when both laser and ultrasound with an intensity of 372 W/cm^2^ are simultaneously transmitted. After this maximum value, light intensity decreased with increasing ultrasound intensity: an increase rate of 15.7 at 435 W/cm^2^ and −6.8 at 496 W/cm^2^. This phenomenon can be explained that the temperature rise due to the incident ultrasound generated coagulation in the laser pathway and the coagulation hindered the forward propagation of light. This coagulation was found even in the case of an ultrasound intensity of 372 W/cm^2^ although the light intensity was the maximum at this ultrasound intensity (Fig. S4). The factor for this temperature rise is inertial cavitation as well as ultrasound intensity itself. This implies that maintaining stable cavitation used in this proposed method is important for achieving the best performance. Note that bubbles are generated but do not collapse in stable cavitation, whereas they suddenly grow and collapse in inertial cavitation^22^. The threshold of ultrasound intensity to stay in stable cavitation depends on the type of tissue. Therefore, it is necessary to investigate the optimal ultrasound intensity for the target tissue before using the proposed method to maximally mitigate the defocusing of incident light and enhance the depth of light penetration.

The ultrasound-assisted light penetration increase is versatile for use in the field of optical imaging and therapy. Theoretically, there are no limitations on the position and size of an ultrasound focal area^16^; thus, an ultrasound transducer can be designed to use it in conjunction with either a tightly focused light beam desirable for optical imaging or a broad uniform beam for photodynamic therapy to increase light penetration. For a deep-lying target region, we can use an ultrasound transducer specially designed for a long depth of focus to generate air bubbles along the light pathway^23^. In general, ultrasound transducers developed for HIFU surgery does not have the desired capability for imaging. Unlike HIFU applications, however, the proposed method does not require a transducer to generate high ultrasound intensity for tissue coagulation. Therefore, it is possible to develop a transducer for ultrasound imaging as well as the ultrasound-assisted light penetration. This feature enables the adoption of the proposed method for ultrasound and optical fusion imaging that has been developed intensively in recent years^24–27^. Another advantage of the proposed method is that any configurations of incident directions of the ultrasound and light are possible if the ultrasound can induce a bubble cloud in the region of interest, which is particularly beneficial for real-time endoscopic applications.

## Methods

### Experimental setup

For the experimental validation of the proposed method, a continuous wave (CW) laser with a wavelength of 660 nm (Nova Pro., RGB Photonics GmbH, Kelheim, Germany) was used as the light source. The laser beam originally had an elliptical shape with a major axis of 0.65 mm and a minor axis of 0.59 mm, and it changed to have a Gaussian profile with a full-width at half-maximum (FWHM) of 3.4 mm after passing through a collimator (F280APC-B, Thorlabs, Inc., Newton, NJ, USA). The laser power module (PowerBox, RGB Photonics GmbH) was responsible for turning on and off the laser source and adjusting the light energy; the laser was transmitted for 10 ms and the peak power was 56 mW. Ultrasound was generated using a ring-shaped 1.1 MHz transducer (H102, Sonic Concepts, Bothell, WA, USA) with outer and inner diameters of 64 and 22.6 mm, respectively. The geometric focal length of this transducer was 62.6 mm. A sinusoid electrical signal was generated using a function generator (AFG3252, Tektronix Inc., Beaverton, OR, USA) with a sampling frequency of 55 MHz. The signal amplitude was increased by 49 dB using a radio-frequency (RF) power amplifier (75A250 A, Amplifier Research Corp., Souderton, PA, USA) to excite the transducer through the RF impedance matching network with a resonance frequency of nearly 1.1 MHz (Fig. S1). The spatial-peak temporal-average intensities of the ultrasound waves generated by changing the electrical signal amplitude to 63.4, 70.6, and 75.6 V were measured^28^ as 200, 268, and 310 W/cm^2^ under the condition that its duty cycle, signal length, and pulse repetition time were maintained to be 53%, 160 ms, and 300 ms.

To examine the air bubbles generated in the phantom at the intensities, a commercial ultrasound scanner (Vantage Research Ultrasound System, Verasonics Inc., Kirkland, WA, USA) was used to acquire ultrasound images of the air bubbles. For this purpose, an ultrasound imaging transducer (C5-2, Verasonics Inc.) was placed in the central opening of the ring-shaped transducer. A detailed description of the experimental setup can be found in *Song et al*.^29^. In the focal area, a bright spot appeared in the ultrasound images^13–15^, indicating the air bubbles generated by the ultrasound transmitted by the ring-shaped transducer.

Both tissue-mimicking phantom and *ex vivo* chicken breast were immersed in a tank filled with degassed, deionized water that was prepared by boiling tap water and subsequently cooling it to room temperature. The focal length of the transducer was placed at a depth of 3 mm below the surface of the sample to induce a bubble cloud in front of the measurement depth, i.e., 8 mm for the phantom and 7 mm for the chicken breast. The laser was delivered into the samples through a hole in the transducer for 20 s (Fig. S1). The light intensity was measured using a CCD camera (CoolSNAP MYO, Photometrics, Tucson, AZ, USA) equipped with an optical lens (Micro-Nikkor 105 mm f/2.8, Nikon Corp., Tokyo, Japan) positioned behind the samples. The camera exposure time was set to 300 ms. The distance between the sample and the lens was maintained at 15 cm. A 660-nm optical filter (FF01-662/11-25, Semrock Inc., Rochester, NY, USA) was used for the measurement. The average normalized intensity within a FWHM of 3.4 mm was calculated after dividing the measurement by the maximum value that the camera could detect (i.e., 2^14^). The experiments on the tissue-mimicking phantom and *ex vivo* chicken breast were repeated 5 times. For the fluorescence imaging experiment, a fluorescent dye (Alexa Fluor 660, Thermo Fisher Scientific Inc., Waltham, MA, USA) of 1 mg ml^−1^ was injected into the chicken breast at a depth of 7 mm. Because this dye emits 683-nm fluorescence after absorbing 660-nm laser, a 683-nm optical filter (FF01-689/23-25, Semrock Inc., Rochester, NY, USA) was placed in front of the CCD camera to measure the fluorescence. The change in light energy at each thickness of the tissue-mimicking phantom was examined using an optical spectrometer (AvaSpec-2048 × 16, Avantes BV, Apeldoorn, The Netherlands); the size of the detector was 3.2 mm in diameter. This experiment was repeated 3 times to verify the increase in the depth of light penetration by the proposed method.

### Sample preparation

The primary component of the tissue-mimicking phantom constructed for the experiments was a degassed water of 77% (v/v), an acrylamide-40% of 18.8% (w/v), an ammonium persulfate of 10% (w/v), a TRIS buffer of 9.72% (v/v), a tetramethylethylenediamine of 0.81% (v/v). While vigorously stirring, a bovine serum albumin (BSA) of 5.5% (w/v) and an intralipid-20% of 1% (v/v) were poured into the mixture solution. The final mixture was hardened at room temperature. For fluorescence imaging experiments, the crescent moon and circle were patterned in the tissue-mimicking phantom (Fig. 5a). The pattern was filled with a mixture of BSA (0.1 mg/ml) and Alexa Fluor 660 (0.1 mg), which was hardened at room temperature. The patterned phantom was placed between the *ex vivo* chicken breast with a thickness of 8 mm and another tissue-mimicking phantom.

## Electronic supplementary material

Supplementary Information
