# Supplementary material for: Increased light penetration due to ultrasound-induced air bubbles in optical scattering media
Source: Sci Rep. 2017 Nov 23;7:16105. doi: 10.1038/s41598-017-16444-9 (PMC5701037; doi:10.1038/s41598-017-16444-9)
Supplement: Supplementary file 1 — Supplementary Information [file 41598_2017_16444_MOESM1_ESM.pdf]

Supplementary Information

Increased light penetration due to ultrasound-induced air bubbles in optical scattering media

Haemin Kim[a], Jin Ho Chang[a,b*]

[a]Department of Biomedical Engineering, Sogang University, 35 Baekbeom-ro, Mapo-gu, Seoul, 04107, South Korea
[b]Department of Electronic Engineering, Sogang University, 35 Baekbeom-ro, Mapo-gu, Seoul, 04107, South Korea

*jhchang@sogang.ac.kr

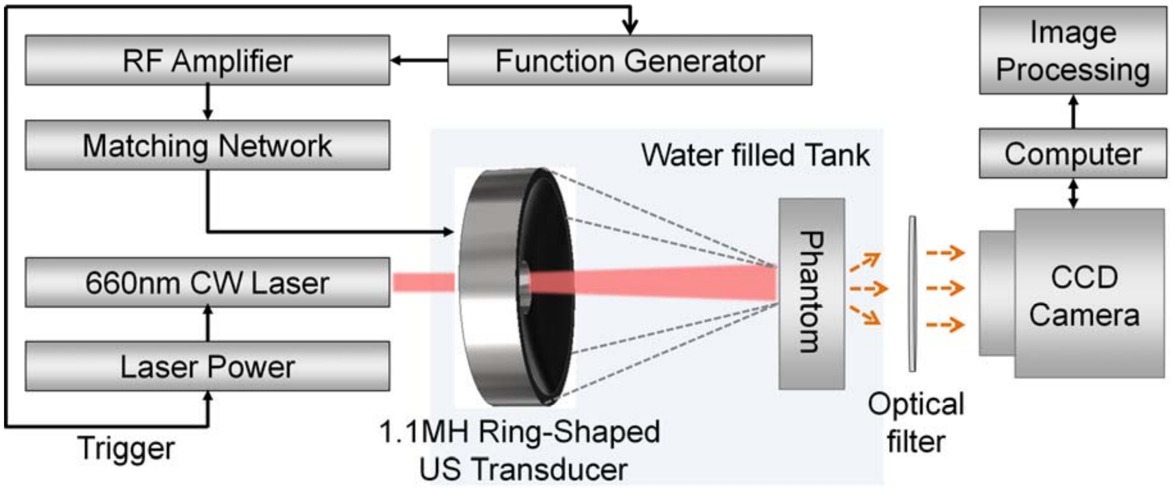

Fig. S1. Detailed experimental arrangement for performance validation of the ultrasound-assisted light penetration increase.

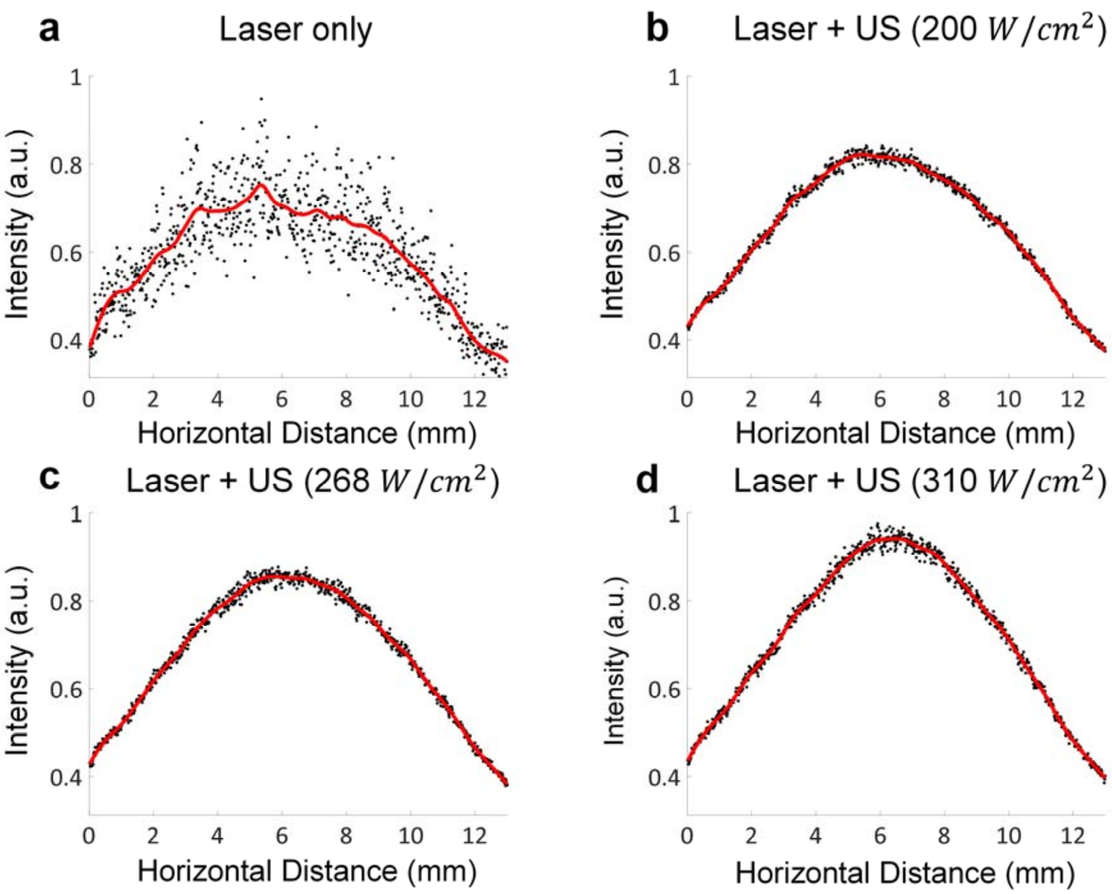

Fig. S2. Light distribution along the horizontal direction measured on the *ex vivo* chicken breast with a thickness of 7 mm, as shown in Fig. 4. The average normalized light intensity and FWHM were 0.68±0.047 and 13.6 mm in the case of (a) laser delivery only, 0.753±0.046 and 13.0 mm, 0.811±0.051 and 12.46 mm, and 0.837±0.390 and 11.76 mm in the cases of the simultaneous delivery of laser and ultrasound with an intensity of (b) 200 W/cm^2, (c) 268 W/cm^2, and (d) 310 W/cm^2.

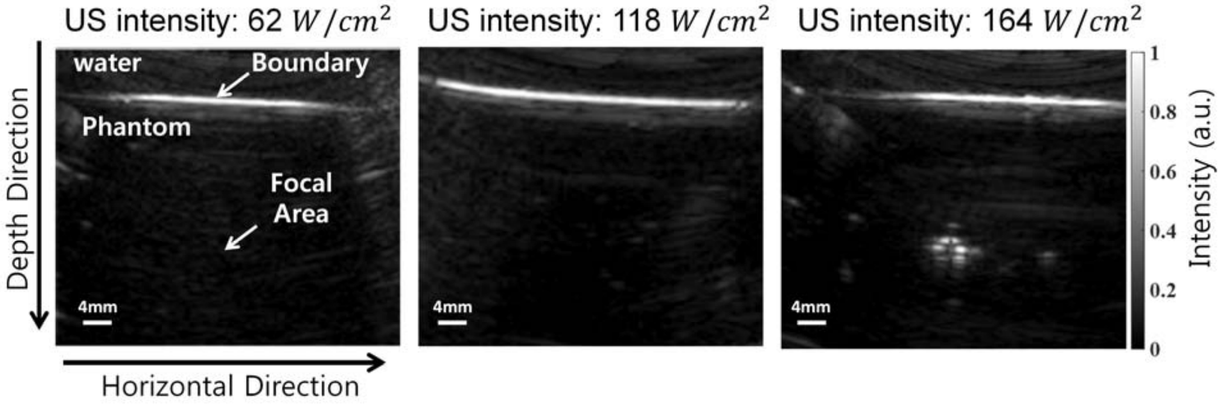

Fig. S3. Ultrasound images acquired after simultaneous transmitting the laser and ultrasound with intensities of 62, 118, and 164 W/cm^2 into the tissue-mimicking phantom. The bubble cloud began to be induced at an ultrasound intensity of 164 W/cm^2.

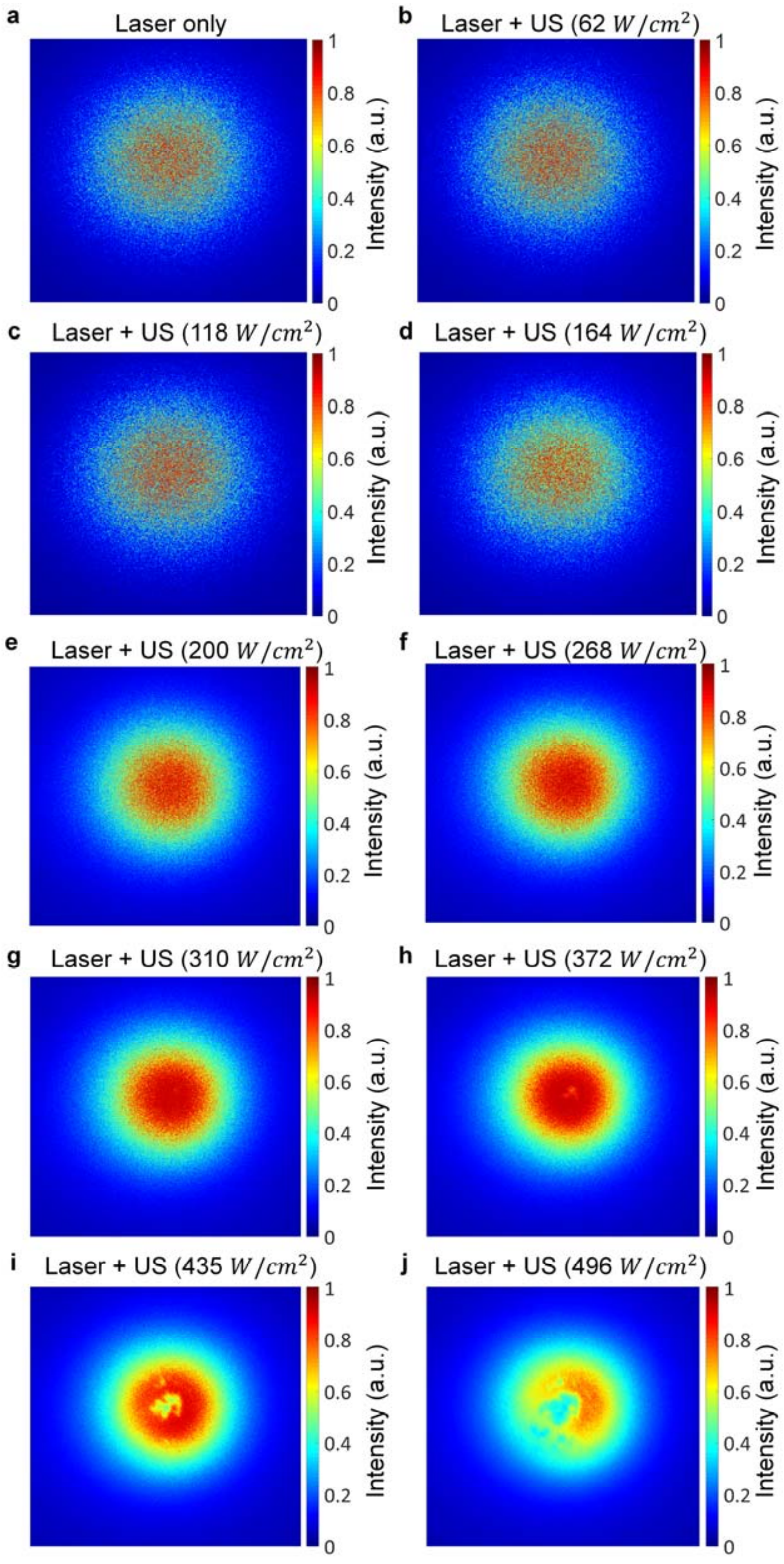

Fig. S4. Measurement of light intensity distribution on the tissue-mimicking phantom with a thickness of 8 mm. (a) When delivering laser only, the average normalized light intensity was 0.594. As the ultrasound intensity was increased to (b) 62, (c) 118, (d) 164, (e) 200, (f) 268, (g) 310, (h) 372, (i) 435, and (j) 496 W/cm^2, the light intensity were changed to 0.599, 0.604, 0.632, 0.669, 0.712, 0.734, 0.744, 0.688, and 0.554. Additionally, a region of low light intensity appeared around the center of the light beam when the ultrasound intensity was (e) 372, (f) 435, and (g) 496 W/cm^2.
